# Antimicrobial Resistance in Extragenital *Neisseria gonorrhoeae* Infections, US Military Centers, 2022–2024

**DOI:** 10.3201/eid3208.260337

**Published:** 2026-08

**Authors:** Jamie L. Dombach, John L. MacArthur, Chothika Mekonnen, Thi Hai Au La, Violet Nxedhlana, Michael H. Norris, Hunter J. Smith, Nathanial K. Copeland, Edwin Kamau

**Affiliations:** William Beaumont Army Medical Center, El Paso, Texas, USA (J.L. Dombach); Tripler Army Medical Center, Honolulu, Hawaii, USA (J.L. Dombach, J.L. MacArthur, C. Mekonnen, N.K. Copeland, E. Kamau); United States Military Academy, West Point, New York, USA (J.L. MacArthur); University of Hawai'i at Mānoa, Honolulu (T.H.A. La, M.H. Norris); University of Hawai'i at Manoa, Manoa, Hawaii, USA (V. Nxedhlana); Armed Forces Health Surveillance Center, Silver Spring, Maryland, USA (H.J. Smith); Uniformed Services University of the Health Sciences, Bethesda, Maryland, USA (H.J. Smith, N.K. Copeland)

**Keywords:** Neisseria gonorrhoeae, antimicrobial resistance, bacteria, sexually transmitted infections, extragenital infection, β-lactamase, surveillance, molecular epidemiology, military population, United States

## Abstract

We analyzed antimicrobial resistance markers in 189 *Neisseria gonorrhoeae* patient encounters from 2 US military medical centers. Co-occurring resistance-associated markers were common; we detected plasmid-mediated β-lactamase in extragenital sites. Our findings highlight the importance of anatomic site–specific surveillance and, potentially, molecular antimicrobial resistance detection to guide screening and treatment strategies.

*Neisseria gonorrhoeae* infection remains a major global public health concern because of increasing antimicrobial resistance (AMR), including reduced susceptibility to extended-spectrum cephalosporins (*1*,*2*). Extragenital infections, particularly in the pharynx and rectum, are often asymptomatic and can contribute to persistence and transmission of resistant organisms (*3*–*5*). 

We analyzed samples from 189 *N. gonorrhoeae*–positive clinical encounters collected during 2022–2024 at Tripler Army Medical Center (TAMC; Honolulu, Hawaii, USA) and Madigan Army Medical Center (MAMC; Tacoma, Washington, USA). The human research protection offices from both institutions, under the umbrella of the Defense Health Agency, reviewed the study and determined it to be research not involving human subjects. Investigators adhered to US policies for the protection of human subjects as prescribed in 45 Code of Federal Regulation 46.

We analyzed data from specimens that included urine and swabs from oropharyngeal, rectal, and urogenital sites (Table). We deidentified data and analyzed it at the encounter level; we were unable to exclude repeat encounters from the same patient. We performed targeted next-generation sequencing using the Urinary Pathogen ID/AMR Panel (Illumina, https://www.illumina.com). We identified AMR markers using predefined sequence-matching rules specific to each gene or mutation (e.g., exact match for point mutations such as *rpsJ* V57M and curated allele grouping for *penA* variants). Because short-read data and panel design did not support reliable allele-level or phylogenetic resolution, we analyzed *penA* variants as a single category. This study did not include phylogenetic reconstruction, multilocus sequence typing, or transmission network analysis; therefore, our findings describe marker prevalence and co-occurrence patterns rather than clonal relationships.

**Table:** Characteristics of patients and specimens in study of antimicrobial resistance patterns in extragenital *Neisseria gonorrhoeae* infections, US military medical centers, 2022–2024*

Characteristic	Encounter site	
	TAMC, n = 101	MAMC, n = 88
Age, y		
Mean	27.0	26.6
Median (range)	24.0 (17–64)	24.0 (19–53)
Sex		
M	84 (83.2)	68 (77.3)
F	17 (16.8)	20 (22.7)
Race		
White	47 (46.5)	35 (39.8)
African American	30 (29.7)	34 (38.6)
Pacific Islander	9 (8.9)	9 (10.2)
Asian	4 (4.0)	0 (0.0)
American Indian	1 (1.0)	2 (2.3)
Other	10 (9.9)	8 (9.1)
Specimen source		
Urine	70 (69.3)	55 (62.5)
Swab	31 (30.7)	33 (37.5)
Oropharyngeal	12 (38.7)	12 (36.4)
Rectal	8 (25.8)	9 (27.3)
Urethra	6 (19.4)	2 (6.1)
Endocervical	4 (12.9)	5 (15.2)
Vaginal	1 (3.2)	4 (12.1)
Genital	0 (0.0)	1 (3.0)

*Values are no. (%) except as indicated. TAMC, Tripler Army Medical Center; MAMC, Madigan Army Medical Center.

Across all encounters, resistance-associated markers frequently co-occurred. The most prevalent markers were *macB* (77.2%) and *rpsJ* V57M (75.7%), and we detected *penA* variants in 63.0% of isolates (Figure). We observed co-occurrence of these markers in 60.3% of encounters, which suggested common resistance-associated marker combinations within the cohort. Findings from TAMC demonstrated higher prevalence of *rpsJ* V57M (84.2% vs. 65.9%; p = 0.004) and *penA* variants (70.3% vs. 54.5%; p = 0.034) than for those from MAMC.

**Figure:**
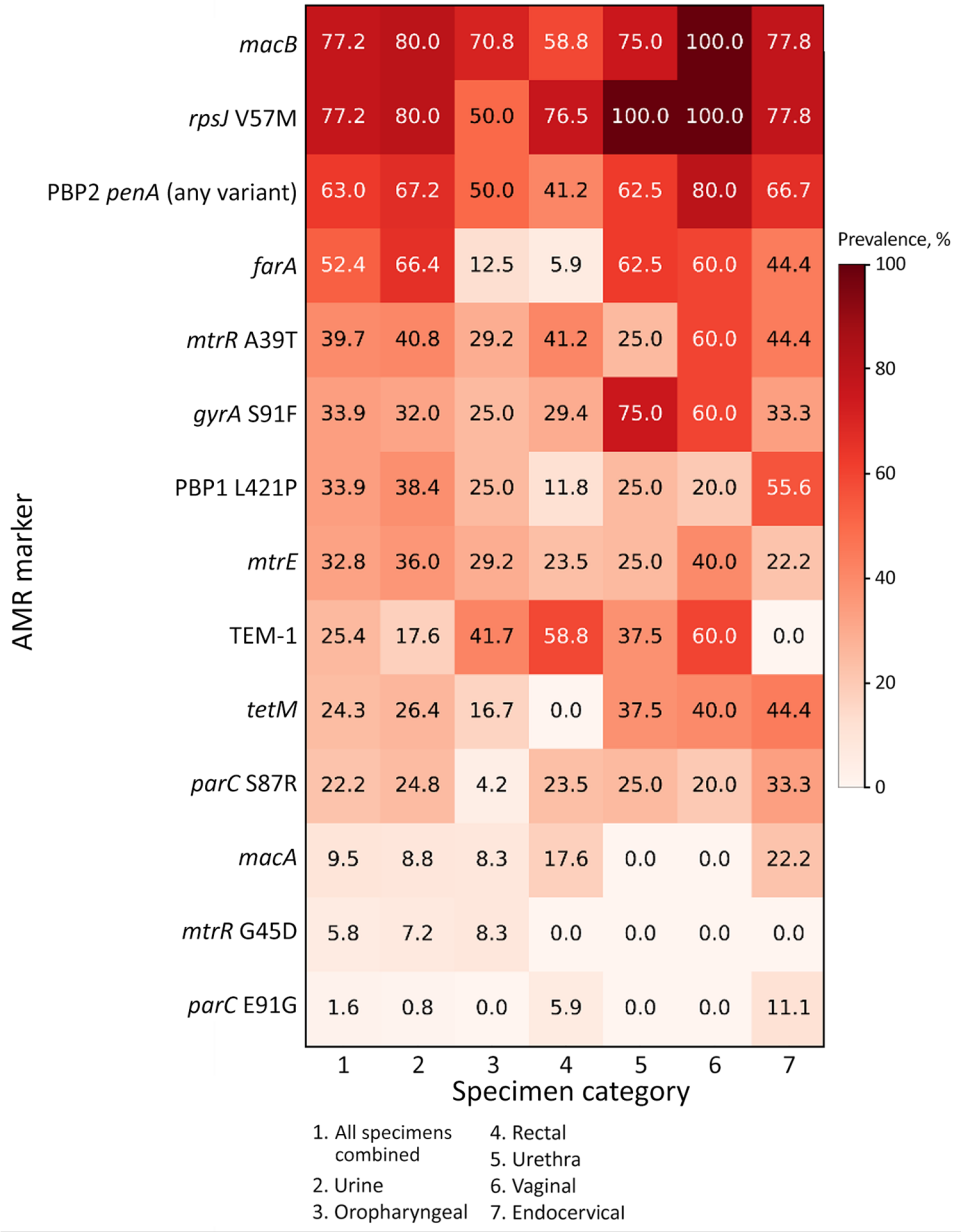
Prevalence of AMR markers in study of AMR patterns in extragenital *Neisseria gonorrhoeae* infections, US military medical centers, 2022–2024. Heatmap shows percent of encounters in which each genotypic marker was detected. Columns indicate specimen categories and rows indicate AMR markers. Values represent percentage positive within each category; color scale reflects prevalence. Detection reflects genotypic presence and does not imply phenotypic resistance. Categories with results <10 should be interpreted cautiously. AMR, antimicrobial resistance.

Markers associated with fluoroquinolone resistance were also present (*gyrA* S91F, 35.4%). We frequently detected structural efflux pump genes (*macB*, *farA*, *mtrE*); however, because those genes are part of the core genome, their presence alone does not predict phenotypic resistance. Regulatory mutations (e.g., *mtrR* variants) and combined genetic contexts are more directly associated with resistance expression.

We observed anatomic site–specific differences. The plasmid-mediated β-lactamase gene *bla*_TEM-1_ was more prevalent in extragenital specimens; we detected *bla*_TEM-1_ in 10/17 (58.8%) of rectal swab samples and 10/24 (41.7%) of oropharyngeal swab samples, compared with 22/125 (17.6%) of urine specimens (Figure). Compared with oropharyngeal swab samples, rectal specimens had increased odds of *bla*_TEM-1_ detection (odds ratio [OR] 6.3; p = 0.004), whereas urine specimens had lower odds (OR 0.29; p = 0.012). In adjusted analyses controlling for age, sex, and institution, rectal specimens showed increased odds and wide 95% CIs (adjusted OR 2.57 [95% CI 0.94–7.00]).

Chromosomal resistance-associated markers such as *rpsJ* V57M were more common in urine specimens (80.0%) than in oropharyngeal specimens (50.0%; OR 3.90; p = 0.014). Conversely, we detected *farA* more frequently in urine (66.4%) than in extragenital (oropharyngeal and rectal) sites (<12.5%) (Figure). Those findings suggest that different anatomic niches may harbor distinct resistance-associated profiles.

Among 189 encounters, 24.9% occurred in persons with documented use of HIV pre-exposure prophylaxis. *bla*_TEM-1_ was more common among users (36.2% vs. 21.8%; p = 0.056), whereas *farA* was less frequent (21.3% vs. 62.7%; OR 0.16, p<0.001). Associations were attenuated after adjustment.

Antimicrobial drug treatment varied across encounters. Doxycycline exposure was common; however, doxycycline is not recommended for treating uncomplicated gonorrhea and was likely prescribed for concurrent or presumptive chlamydial infection or syndromic management (*6*). A high (78.3%) percentage of isolates exposed to doxycycline contained *rpsJ* V57M, consistent with tetracycline-associated resistance markers (*7*).

Our study targeted sequencing and short-read data, which limited resolution of gene variants (e.g., *bla*_TEM_ subtypes) and precluded plasmid reconstruction or phylogenetic analyses. The absence of phenotypic susceptibility testing limited our ability to interpret clinical resistance. Retrospective data and incomplete sampling across anatomic sites might affect prevalence estimates. Finally, lack of temporal and patient-level linkage data limited inferences regarding transmission dynamics.

In summary, we identified frequent co-occurrence of resistance-associated markers in *N. gonorrhoeae* from US military medical centers and found enrichment of *bla*_TEM-1_ in extragenital sites. Our findings support the value of anatomic site–specific surveillance, especially for extragenital infections in sexually transmitted infections, and molecular AMR marker detection (*1*,*4*,*8*,*9*).
